# Supplementary material for: Seasonal shift in timing of vernalization as an adaptation to extreme winter
Source: eLife. 2015 Jul 23;4:e06620. doi: 10.7554/eLife.06620 (PMC4532801; doi:10.7554/eLife.06620)
Supplement: Figure 2—source data 1. — DOI: http://dx.doi.org/10.7554/eLife.06620.007 [file elife06620s001.pdf]

Figure 2 – source data 1

Primers used for qPCR ChIP.

Primers	Sequence (5'-3')
-2429_F<br>-2376_R	ATCCAGAAAAGGGCAAGGAG<br>CGAATCGATTGGGTGAATG
-1708_F<br>-1639_R	TGGAGGGAACAACCTAATGC<br>TCATTGGACCAAACCAAACC
-501_F<br>-381_R	ACTATGTAGGCACGACTTTGGTAAC<br>TGCAGAAAGAACCTCCACTCTAC
-158_F<br>-56_R	GCCCGACGAAGAAAAAGTAG<br>TCCTCAGGTTTGGGTTCAAG
307_F<br>393_R	GGCGGATCTCTTGTTGTTTC<br>CTTCTTCACGACATTGTTCTTCC
543_F<br>700_R	CGTGCTCGATGTTGTTGAGT<br>TCCCGTAAGTGCATTGCATA
1424_F<br>1561_R	TTGACAATCCACAACCTCAATC<br>TCAATTTCCTAGAGGCACCAA
2356_F<br>2451_R	AGTTTGGCTTCCTCATACTTATGG<br>CAATGAACCTTGAGGACAAGG
3088_F<br>3224_R	GGGGCTGCGTTTACATTTTA<br>GTGATAGCGCTGGCTTTGAT
4213_F<br>4360_R	AGAACAACCGTGCTGCTTTT<br>TGTGTGCAAGCTCGTTAAGC
5030_F<br>5135_R	CCGGTTGTTGGACATAACTAGG<br>CCAAACCCAGACTTAACCAGAC
6768_F<br>6838_R	TTGTAAAGTCCGATGGAGACG<br>ACTCGGCGAGAAAGTTTGTG
STM exon F<br>STM exon R	GCCCATCATGACATCACATC<br>GGGAACTACTTTGTTGGTGGTG
