# Supplementary material for: Seasonal shift in timing of vernalization as an adaptation to extreme winter
Source: eLife. 2015 Jul 23;4:e06620. doi: 10.7554/eLife.06620 (PMC4532801; doi:10.7554/eLife.06620)
Supplement: Figure 3—source data 1. — DOI: http://dx.doi.org/10.7554/eLife.06620.010 [file elife06620s002.pdf]

Figure 3 – source data 1

Developmental stage of natural *Arabidopsis thaliana* populations in spring in the High Coast area of N. Sweden (62.5^{o}N)

Pop.	Sampling	RW	RWO	New germinants
Ådal	T 13	58	0	0
Högsjö	T 20	260	2*	0
Alnö, Hovid I	A	50	0	0
Alnö, Hovid II	A	55	0	0
Eden, road I	A	63	1+5*	0
Eden, road II	A	31	1#	0
Lövvik	T 4	39	0	0
Lövvik	A	285	1+3#	0
Total		841	13*#	0

- partly decomposed and dying plants

located under other plants

Number of *Arabidopsis thaliana* rosettes with visible buds or inflorescence (RW), rosettes without a visible bud or inflorescence (RWO), newly germinated seedlings. Plants were assessed every 0.5 m along a transect (T n) in squares (14.5 cm x 19.5 cm) or in an area (A) of ~ 1 m^2. Scoring was undertaken five to six weeks after snowmelt in natural populations in the High coast area of Sweden (2-6th May 2015).

Population size was ~2000 – 3000 in both Ådal and Högsjö, 1000-2000 in Lövvik and all plants were recorded in Eden and the main part in Hovid.

A transect was possible to set up in Ådal and Högsjö but not in Eden and Hovid.
