# Supplementary material for: Seasonal shift in timing of vernalization as an adaptation to extreme winter
Source: eLife. 2015 Jul 23;4:e06620. doi: 10.7554/eLife.06620 (PMC4532801; doi:10.7554/eLife.06620)
Supplement: Figure 4—source data 1. — DOI: http://dx.doi.org/10.7554/eLife.06620.019 [file elife06620s003.docx]

**Figure 4 – source data 1**

Cabinet flowering time data were selected where conditions most closely matched mean temperatures recorded during 2011 and 2012 field experiments.

| **2011** | | | | | |
| --- | --- | --- | --- | --- | --- |
| Field Temperatures | | | | | |
| Weeks after sowing | 4 | 5 | 6 | 8 | 12 |
| Cumulative  average  temperature | 12.48^o^C | 10.82^o^C | 9.72^o^C | 8.40^o^C | 6.42^o^C |
| Selected Cabinet Data | | | | | |
| Weeks vernalization | 4 | 5 | 6 | 8 | 12 |
| Constant temperature | 12^o^C | 10^o^C | 8^o^C | 8^o^C | 5^o^C |

| **2012** | | | | |
| --- | --- | --- | --- | --- |
| Field Temperatures | | | | |
| Weeks after sowing | 4 | 6 | 8 | 12 |
| Cumulative  average  temperature | 9.49^o^C | 7.34^o^C | 5.61^o^C | 4.55^o^C |
| Selected Cabinet Data | | | | |
| Weeks vernalization | 4 | 6 | 8 | 12 |
| Constant temperature | 8^o^C | 8^o^C | 5^o^C | 5^o^C |
